# Treatment of the Asphyxia of New-Born Children

**Published:** 1857-11

**Authors:** Marshall Hall


*Treatment of the Asphyxia of New-born Children.*—By Dr. Marshall Hall.

"The treatment of the still-born infant may finally be thus briefly resumed in the form of rules:

"1st. Place the fœtus on the face.

"2d. Sprinkle the general surface briskly with cold water.

"3d. Make gentle pressure on the back; remove it, and turn the infant on the side, and again place it prone with pressure.

"4th. Rub the limbs, with gentle pressure, upwards.

"5th. Repeat the sprinkling only now, with cold and hot water, (of the temperatures of 60° and 100° Farhn. alternately.)

"6th. Continue these measures, or renew them, from time to time, even for hours. The embers of life may not be enirely extinct.—*Med. Times & Gazette.*

